# Resection of a meningioma, vestibular schwannoma, and placement of auditory brainstem implant using translabyrinthine approach

**DOI:** 10.3171/2021.7.FOCVID2163

**Published:** 2021-10-01

**Authors:** Nickalus R. Khan, Clifford S. Brown, Simon Angeli, Jacques J. Morcos

**Affiliations:** Departments of 1 Neurosurgery and; 2 Neurotology, University of Miami Miller School of Medicine, Miami, Florida

**Keywords:** vestibular schwannoma, acoustic schwannoma, surgery, auditory brainstem implant, meningioma, neurofibromatosis, hearing

## Abstract

The authors present the case of a 34-year-old patient with neurofibromatosis type 2 (NF-2) who underwent a left translabyrinthine approach for resection a meningioma, vestibular schwannoma, and placement of an auditory brainstem implant (ABI). They review the preoperative workup, technical nuances of the surgery, and cadaveric dissections with anatomical diagrams, and provide a review on ABIs. The patient remained neurologically intact and had improvement in lip reading when using the ABI device in the postoperative period.

The video can be found here: https://stream.cadmore.media/r10.3171/2021.7.FOCVID2163

## Transcript

### 0:29 Clinical Presentation and Neurological Examination

A 34-year-old female with NF-2 and multiple tumors, losing hearing on the left side that has failed Avastin as well as twice Gamma Knife to the left tumor.

### 0:43 Preoperative Audiogram

Here is the audiogram showing anacusis on the left side.

### 0:51 Neuroimaging

One can appreciate on MRI the multiple meningiomas and the bilateral acoustic neuromas. The lesion of interest is on the left side.

### 1:12 Rationale for the Procedure

The rationale for doing the procedure is the complete loss of hearing on the left side, tumor growth, failure of previous radiosurgery twice and her young age with an opportunity to train her on the ABI before she loses hearing in the right hear.

### 1:23 Risks of the Procedure and Potential Benefits

Risks of the procedure include infection, hematoma, facial nerve weakness, incorrect placement of the ABI, occasionally death. The benefits are of course tumor control and possibly hearing restoration to the left ear.

### 1:36 Alternatives for Treatment

The alternatives do not include radiosurgery anymore because she has already had the procedure performed twice.

### 1:45 Description of Setup

The setup includes supine position with a shoulder bump for a translabyrinthine approach with the head turned 45°. The necessary equipment includes the usual neuro-otologic and neurosurgical equipment as well as intraoperative monitoring.

### 2:11 Completed Mastoidectomy

The translabyrinthine approach is shown here having been well underway on the left side. The neuro-otologist performs this part of the procedure.

### 2:42 Sigmoid Sinus Decompression

The sigmoid sinus can be decompressed further both presigmoid and retrosigmoid.

### 2:47 Sinodural Angle

The sinodural angle is being exposed here at the junction between the middle and the posterior fossa. Diamond drilling is being used close to the dura after using the cutting drill earlier in the drilling.

### 3:04 Retrosigmoid Decompression

The retrosigmoid decompression is important particularly when there are large tumors.

### 3:12 Labyrinthectomy

The labyrinthectomy now is underway particularly with the superior semicircular canal here being delineated in a circular fashion. The fallopian canal is delineated. The lateral semicircular canal, the lumen of which is well seen. The sigmoid sinus is mobilized posteriorly and here is the presigmoid dura with the endolymphatic duct and endolymphatic sac visualized. One can appreciate the location of Donaldson’s line.

### 3:57 Inferior Trough IAC

Now as we are approaching the internal auditory canal the inferior trough is being delineated and drilled. The jugular bulb is noted and is not particularly high in this case.

### 4:33 Superior Trough IAC

This is the superior trough of the internal auditory canal being delineated with the diamond drill bit.

### 6:53 Anatomical Drawing of Cerebellopontine Angle

Here is a dissection and a schematic from Rhoton to remind us where choroid plexus, flocculus, and lateral recess of the fourth ventricle are.

### 7:21 Creation of Pocket for ABI Receiver

We are here placing the ABI receiver in a subcutaneous pocket.

### 7:39 Placement of Auditory Brainstem Implant

Here is the electrode end of the ABI.

### 7:49 Anatomical Drawing of Foramen of Luschka

Again, the goal is to enter the lateral recess of the fourth ventricle to place it on top of the cochlear nuclei area through the foramen of Luschka. Naturally the correct surface of the electrode has to be placed down on the brainstem floor.

### 8:18 Anatomical Drawing of the Floor of the Fourth Ventricle

Here is a schematic of where the various nuclei are in the floor of the fourth ventricle overlaid on a Rhoton dissection.

### 8:57 Confirmation of Correct Placement

The audiology team will then confirm correct placement by interrogating a pair of electrodes and to make sure we are on top of the cochlear area.

### 9:02 Disease Background

ABI is the most common placed surface stimulator in the CNS. It was first developed in the 1970s at the House Ear Institute.

### 9:12 Review of Clinical and Imaging Outcome

The patient remained neurologically intact. She currently has 15 out of the 21 electrodes. Two of the electrodes are turned off because of a sensation in her throat. Four of them are turned off because they are stimulating probably the medial longitudinal fasciculus. She has improved significantly in her lip-reading ability when her implant is turned on. A postoperative MRI showed essentially gross-total resection of the acoustic neuroma.

### 9:44 References^1–4^

## Disclosures

The authors report no conflict of interest concerning the materials or methods used in this study or the findings specified in this publication.

## Author Contributions

Primary surgeon: Morcos, Angeli. Assistant surgeon: Khan, Brown. Editing and drafting the video and abstract: Morcos, Brown. Critically revising the work: Morcos, Brown, Angeli. Reviewed submitted version of the work: Morcos, Brown. Approved the final version of the work on behalf of all authors: Morcos. Supervision: Morcos.
